# Fermented botanical product supports lignin development and suppresses soil-borne pathogens

**DOI:** 10.1186/s13104-026-07716-7

**Published:** 2026-02-17

**Authors:** Yuri Mizuno, Akiko Hida, Chika Tadokoro, Junichi Kato, Yasuhide Asano, Yusuke Tateuchi, Kotaro Fujioka, Shinsuke Kishida, Hideto Torii

**Affiliations:** 1 Department of Research and Development, Manda Fermentation Co., Ltd, 5800-121 Innoshima Shigei-cho, Onomichi, Hiroshima, 722-2102 Japan; 2 Program of Biotechnology, Graduate School of Integrated Sciences for Life, Hiroshima University, 1-3-1 Kagamiyama, Higashi-Hiroshima, Hiroshima, 739-8530 Japan

**Keywords:** *Ralstonia pseudosolanacearum*, *Clavibacter michiganensis* subsp. *michiganensis*, Fermented botanical product, Lignin

## Abstract

**Objective:**

Soil-borne plant pathogens are a significant cause of economic loss in global agriculture. In a previous study, fermented botanical product (FBP) treatment was shown to suppress bacterial wilt disease caused by *Ralstonia pseudosolanacearum* (*Rps*) in tomatoes. This study evaluated whether treatment with FBP suppresses soil‑borne bacterial pathogens and induces host plant defense responses.

**Results:**

Seedlings of three Solanaceae species and nine families of plants treated with water or FBP for 48 h were inoculated with *Rps* and wilting symptoms were compared 14 days post inoculation. FBP treated plants showed an increase in the number of individuals without wilting symptoms and a decrease in the number of dead individuals. In addition, inoculation with *C. michiganensis* subsp. *michiganensis*, which belongs to the same soil-borne bacterial disease group, showed similar suppression in tomatoes treated with FBP. To further confirm the root defense response to FBP treatment, we analyzed the amount of lignin, which is known to be a physical barrier against pathogens, and found that FBP application increased the amount of lignin in Solanaceae plants. These results suggest that FBP suppress soil-borne bacterial diseases by supporting lignin development.

**Supplementary Information:**

The online version contains supplementary material available at 10.1186/s13104-026-07716-7.

## Introduction

Potential yield loss caused by plant pathogens is estimated to be as high as 16% worldwide [1]. The rhizosphere contains up to 10^6^–10^9^ bacteria/g and is constantly attacked by soil-borne pathogens [2, 3]. The soil-borne bacterium *Ralstonia pseudosolanacearum* (*Rps*), which causes bacterial wilt, is a major pathogen of tomatoes [4], infecting > 200 plant species from > 53 families, including Solanaceae [5]. Pathogenic bacteria enter root vascular bundles and travel through the vascular system to stem and aerial parts of the plant, where they multiply rapidly and cause wilting symptoms [6, 7]. Fermented botanical product (FBP) are eco-friendly materials produced by fermenting natural raw materials, including fruits, grains, vegetables, seaweed, and sugars. We showed that FBP exert concentration-dependent control against bacterial wilt caused by *Rps* in tomatoes grown in FBP-treated soil; furthermore, few or no bacterial cells were detected in aerial parts of the tomato, despite the absence of toxicity in FBP, suggesting that FBP may control the disease by activating plant immunity in the roots [8]. While there is evidence that plant-derived fermented extracts and fermentation products exhibit antibacterial and biocontrol activity against soil-borne pathogens, including *Ralstonia* spp., there is a paucity of published plant-targeted studies on FBP used in this study [9]. According to the extant literature, Hida et al. is the sole published study that has investigated the effects of FBP on plants [8]. However, it remains unclear whether FBP can suppress bacterial wilt disease in hosts other than tomatoes or whether they are effective against other soil-borne pathogens. The mechanism by which plant immunity is activated is also unclear.

Resistant cultivars are used to control bacterial wilt disease in multiple plant species, and differences in the infection process with susceptible cultivars have been studied [6]. Plants possess physical barriers and inducible defense mechanisms against pathogens [10]. Cell walls are the primary barrier against pathogen invasion, and studies have documented alterations in the expression of cell wall-related genes, such as lignin, in resistant cultivars in response to bacterial invasion [11].

In this study, we evaluated the efficacy of FBP in controlling bacterial wilt disease and bacterial canker of tomato. Additionally, we focused on root lignin to determine the effects of FBP treatment on root immunity and investigate its potential to enhance plant physical barriers.

## Materials and methods

### Overview of FBP and its compositional profile

As previously described [8], FBP was made from 41 types of raw materials (fruits, vegetables, grain crops, and edible algae, including brown sugar, and honey). The product underwent static fermentation at room temperature with occasional stirring for 3 years without the addition of water. Following fermentation, filtered through a strainer to remove solids.

### Plant material and growth conditions

The plants used in this study are listed in Table 1. Seeds were sterilized with 8.75% (v/v) sodium hypochlorite and 0.1% Tween 20 for 10 min and washed with sterilized water for 90 min. Plants were grown on a 50:50 mixture of Super Mix A (Sakata seed, Japan) and Nippi Engei-Baido 1 (Nihon Hiryo, Japan) in a growth chamber under a 16 h light (23 °C or 28 °C)/8 h dark (23 °C) cycle for different durations (Table 1).

**Table 1:** Bacterial strains and conditions for inoculation assay in this study

Family	Host plants	Manufacturer	Cultivation days to inoculation (days)	Pathogenic bacteria	Optical density at 600 nm	Volume (ml)
	Species			Bacterial strains		
Solanaceae	*Capsicum annuum* ‘*grossum*’. cv. California Wonder	Matsuo Farm	21	*R. pseudosolanacearum* MAFF 106611	0.3	5.0
Solanaceae	*Solanum melongena * cv. SenryoNigou	TAKII & CO.,LTD.	21	*R. pseudosolanacearum* MAFF 106611	0.3	3.0
Solanaceae	*Capsicum annuum* cv. Nagano	TAKADA SEED Co., LTD.	21	*R. pseudosolanacearum* MAFF 106611	0.3	3.0
Brassicaceae	*Brassica rapa* subsp. *Rapa* cv. Kanamachikokabu	TAKII & CO.,LTD.	12	*R. pseudosolanacearum* MAFF 106611	1.0	1.0
Asteraceae	*Glebionis coronaria* cv. Nakaha Shungiku	TAKII & CO.,LTD.	12	*R. pseudosolanacearum* MAFF 106611	1.0	1.0
Plumbaginaceae	*Limonium sinuatum* cv. Elliot mix	SAKATA SEED CORP.	28	*R. pseudosolanacearum* MAFF 106611	1.0	1.0
Ranunculaceae	*Delfinium grandiflorum* cv. aurora mix	SAKATA SEED CORP.	21	*R. pseudosolanacearum* MAFF 106611	1.0	1.0
Rosaceae	*Fragaria* × *ananassa* cv. Yotsuboshi	Mie Kono Co., Ltd.	28	*R. pseudosolanacearum* MAFF 311121	1.0	2.0
Portulacaceae	*Portulaca grandiflor* cv. Solar kids	SAKATA SEED CORP.	56	*R. pseudosolanacearum* MAFF 331016	1.0	1.0
Linaceae	*Linum usitatissimum*	Greenfield Project Inc.	12	*R. pseudosolanacearum* MAFF 106611	0.5	5.0
Malvaceae	*Malva verticillata*	TAKII & CO.,LTD.	11	*R. pseudosolanacearum* MAFF 106611	0.5	5.0
Convolvulaceae	*Calonyction aculeatum* cv. Shirobana yugao	SAKATA SEED CORP.	11	*R. pseudosolanacearum* MAFF 106611	0.5	5.0
Solanaceae	*Solanum lycopersicum* cv. Ogatafukuju	Marutane CORP.	12	*C. michiganensis* subsp. *michiganensis* MAFF301516	0.3	1.0

### Infection assay

Bacterial strains were obtained from the National Agriculture and Food Research Organization, cultured on casamino acid–peptone–glucose (CPG) medium for *Rps* and yeast–peptone–glucose–sucrose (YPGS) medium for *C. michiganensis* subsp. *michiganensis* (*Cmm*), incubated at 28 °C for 3 days. Information on the bacterial strains used in this study is summarized in Table 1. The plant infection assay was performed as previously described [8]. Briefly, after soaking for 2 days in H_2_O or FBP, the roots were wounded using scalpels. Cultivated bacteria were washed twice with sterilized water. Bacterial suspensions and growing days to infection were prepared according to the conditions indicated in Table 1, and inoculated around wounded plants. Disease symptoms were evaluated on a five-point scale, with wilting as an indicator.

### Lignin analysis

Root lignin content was measured using the previously reported method [12]. Lignin was extracted using 20 mg of ground root. The supernatant was dissolved in 1 N NaOH, and absorbance was measured at 280 nm.

### Growth assay for cellulase activity of *Cmm*

For the growth assay, samples were cultured in 1/2 YPGS medium and inoculated with or without 0.5% FBP to a final optical density at 600 nm (OD_600_) of 0.01. After 24 and 48 h of cultivation, OD_600_ was measured. Cellulase activity of was measured using a previously reported method [13]. Bacteria cultured in YPGS medium with or without 0.5% FBP were inoculated on M9CMC (M9 medium, 0.1% yeast extract, 0.5% carboxymethylcellulose). Plates were incubated for 7 days at 28 °C. Plates were stained with 0.1% Congo red for 20 min and bleached with 1 M NaCl.

### RNA isolation and sequencing

Leaves and roots of tomato plants treated with water or FBP for 2 days were collected and frozen in liquid nitrogen. Total RNA was extracted using a commercial kit and used to prepare strand‑specific cDNA libraries with polyA selection. Libraries were sequenced on the DNBSEQ platform (BGI Genomics) and clean reads were aligned to the *S. lycopersicum* reference genome (GCF_000188115.4_SL3.0) for gene quantification and differential expression analysis using HISAT [14]. Full experimental and bioinformatics details are provided in Supplementary Methods.

## Results

### Effect of FBP treatment on bacterial wilt in various plants

To elucidate the impact of FBP treatment on bacterial wilt disease suppression in plants other than tomatoes, the infection conditions in a selection of test plants were examined with reference to [15].

**Fig. 1:**
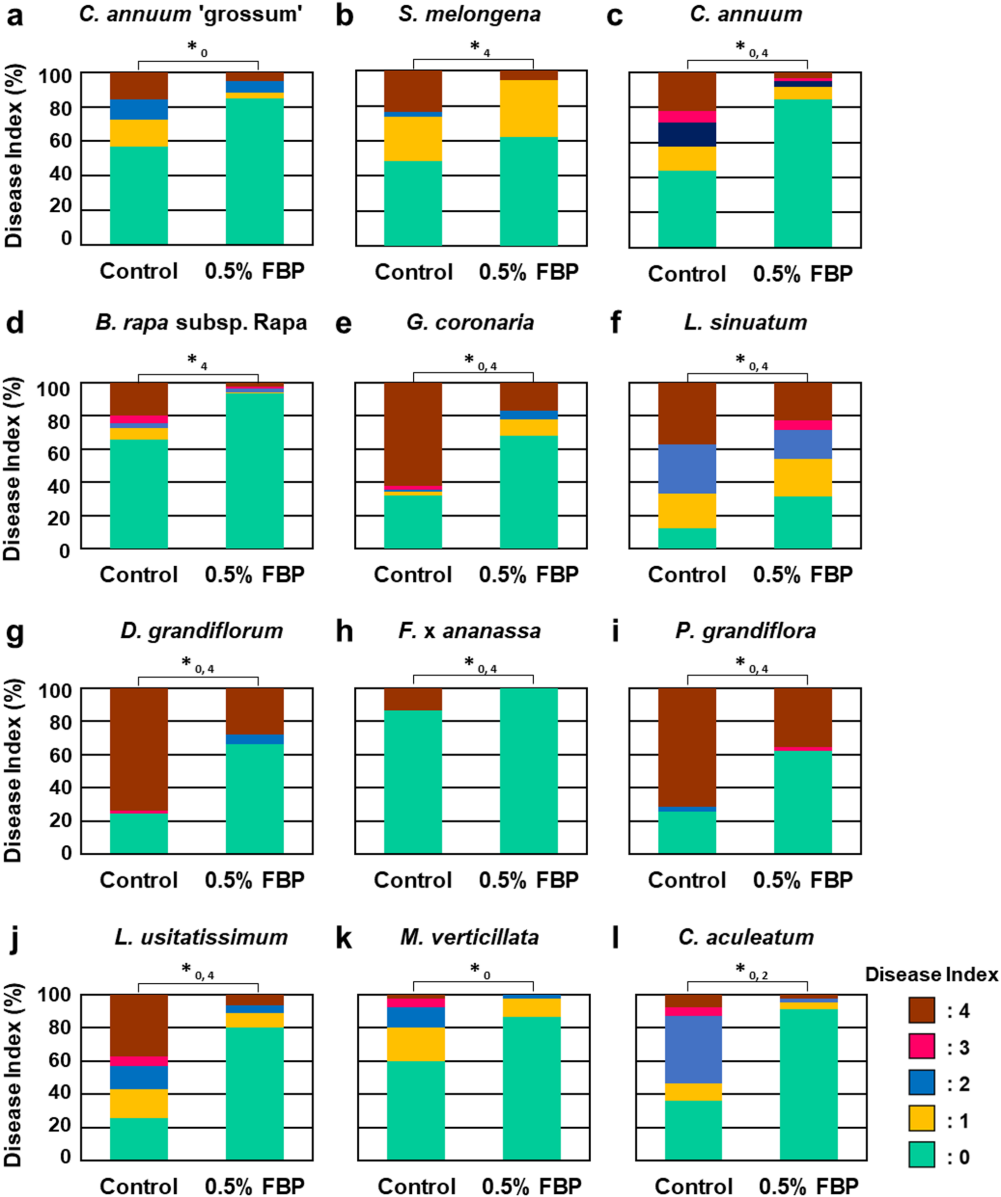
Infection assay of *Rps* on plants. **a–l** Host plants were inoculated with *Rps* and appearance of disease symptoms was categorized into 5 classes according to the severity of disease symptoms. 0, no visible symptom; 1, > 25% of leaves wilting; 2, > 50% of leaves wilting; 3, > 75% of leaves wilting; 4, greater than 75% wilting or dead. Plot showing percentage of plants with disease symptom severities represented in the five classes 14 days post-inoculation. Total of 15–30 plants were prepared for each experiment, with at least three experiments being conducted. Data marked with asterisks are significantly different from control as assessed by Pearson’s Chi-square test: *P < 0.05

*Rps* was inoculated under the conditions listed in Table 1. Wilt symptoms were evaluated 14 days after inoculation using a five-point scale. FBP treatment confirmed a significant difference in the ratio of index 0 for *C. annuum* ‘grossum’ and *M. verticillate*, index 4 for *S. melongena* and *B. rapa* subsp. Rapa, and indices 0 and 2 for *C. aculeatum* (Fig. 1). Seven species exhibited substantial variation in indices 0 and 4. These results suggest that FBP is effective in controlling bacterial wilt disease not only in Solanaceae plants, but also in nine other families.

### Effect of FBP treatment on bacterial canker in tomato

**Fig. 2:**
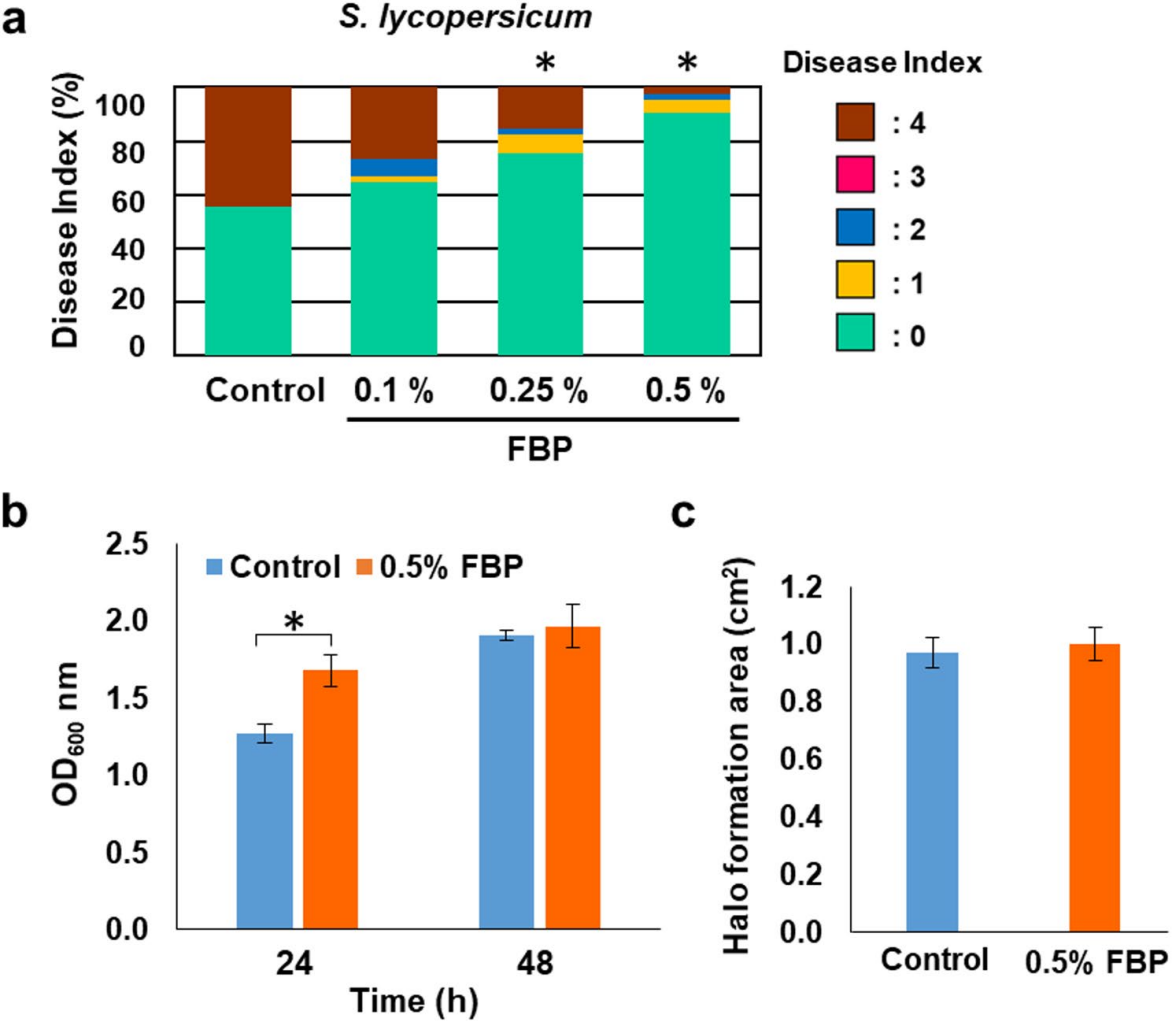
Effect of FBP on *Cmm*. **a** Tomato plants that had soaked in water as a control and in 0.5% FBP for 48 h were inoculated with Cmm. Plot showing percentage of plants with disease symptom severities represented in the five classes 21 days post-inoculation. A total of fifteen plants were prepared for each experiment, with three experiments being conducted. Data marked with asterisks are significantly different from control as assessed by Pearson’s chi-square test: *P < 0.05. **b** Growth in 1/2 YPGS liquid medium ± 0.5% FBP. Means ± SE (*n* = 3). Data marked with asterisks are significantly different from control as assessed by Student t-test. **c** Halo area was observed after a 5-day incubation with M9CMC medium. Halo area was measured using ImageJ. Means ± SE (*n* = 3)

To investigate the effectiveness of FBP-treated plants in controlling pathogens other than *Rps*,* Cmm*, a known soil-borne bacterium, was inoculated on tomatoes treated with varying concentrations of FBP. At 21 days after inoculation with *Cmm*, concentration-dependent suppression of wilt symptoms was observed in FBP-treated tomatoes, with 0.25% and 0.5% FBP treatments being significantly effective in suppressing these symptoms (Fig. 2a). FBP have no antibacterial effects on *Rps* [8]. To confirm that FBP did not have toxic effects on *Cmm*, bacterial growth was evaluated in the presence of FBP in a liquid medium. FBP addition promoted pathogen growth, and these cells did not exhibit toxicity (Fig. 2b). Cellulase genes are a major virulence factors in pathogens, and are associated with wilt symptoms [16]. To evaluate the virulence of the bacteria in the presence of FBP, they were cultured in a medium containing cellulose; cellulase activity was evaluated based on the halo area. The pathogen cultivated in 0.5% FBP did not influence cellulase activity on the medium (Fig. 2c).

### Effect of FBP treatment on lignification of Solanaceae plants

**Fig. 3:**
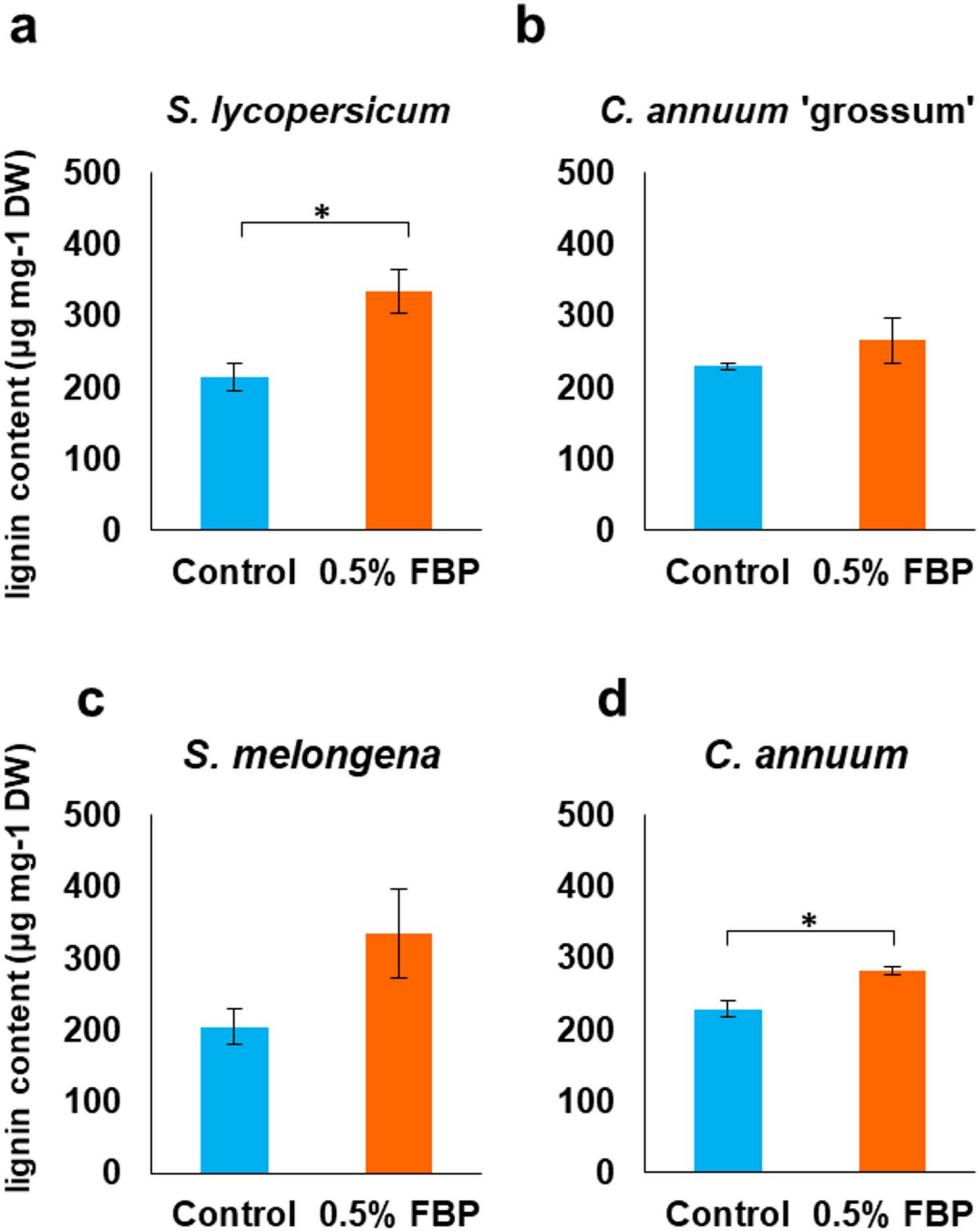
Lignin content of Solanaceae plants.** a**–**d** Lignin content in tomato and paprika roots. Plants that had soaked in water as a control and in 0.5% FBP for 48 h and 17 days-old (*S. lycopersicum*) and 24-days-old (*C. annuum* ‘grossum’, *S. melongena*, *C. annuum*) roots were collected and determined as thioglycolic acid lignin. Means ± SE (*n* = 3). Data marked with asterisks are significantly different from control as assessed by the Student’s t-test: *P < 0.05

FBP treatment leads to few or no *Rps* cells in aerial parts of tomato plants [8]. Therefore, we investigated the potential of FBP treatment in enhancing root immunity by focusing on lignin, a known barrier against plant pathogens. The roots of Solanaceae plants were collected after 5 days of treatment with water or 0.5% FBP for 48 h and quantified for lignin content. FBP-treated plants showed an increase in root lignin content, which was particularly significant in *S. lycopersicum* and *C. annuum* (Fig. 3a–d). Lignin is synthesized via the phenylpropanoid pathway, commencing with phenylalanine [17]. Therefore, we performed RNA-seq analysis and extracted DEGs associated with the phenylpropanoid pathway to confirm the effect of FBP treatment on tomato root genes; 19 of the 23 DEGs detected were overexpressed by more than two-fold following FBP treatment, including upstream phenylalanine ammonia lyase (*PAL*) genes and downstream peroxidase genes (Table 2).

**Table 2:** Tomato root genes with differentially expressed genes in the phenylpropanoid pathway

Gene ID	Gene symbol	Root_Control Average TPM	Root_FBP average TPM	log2 (fold change)	Q value	KEGG_A_class	KEGG pathway description
101246969	‘LOC101246969’	0.4	20.6	5.9	0.01	Phenylalanine ammonia-lyase-like	00360///Phenylalanine metabolism+++00940///Phenylpropanoid biosynthesis
101260610	‘LOC101260610’	0.0	1.8	5.4	0.00	Acetyl-CoA-benzylalcohol acetyltransferase	00940///Phenylpropanoid biosynthesis+++00941///Flavonoid biosynthesis+++00945///Stilbenoid, diarylheptanoid and gingerol biosynthesis
101248494	‘LOC101248494’	1.8	56.3	5.0	0.00	Cationic peroxidase 1	00940///Phenylpropanoid biosynthesis
101253989	‘LOC101253989’	0.8	11.1	3.8	0.00	Phenylalanine ammonia-lyase-like	00360///Phenylalanine metabolism+++00940///Phenylpropanoid biosynthesis
101261765	‘LOC101261765’	1.9	13.3	3.0	0.03	Cytochrome P450 98A3	00940///Phenylpropanoid biosynthesis+++00941///Flavonoid biosynthesis+++00945///Stilbenoid, diarylheptanoid and gingerol biosynthesis
101256157	‘LOC101256157’	2.4	16.6	2.9	0.00	Anthocyanidin 3-O-glucosyltransferase 5	00940///Phenylpropanoid biosynthesis
101261825	‘LOC101261825’	7.9	54.7	2.8	0.02	Peroxidase P7	00940///Phenylpropanoid biosynthesis
101245815	‘TMP1’	7.4	50.0	2.8	0.01	Suberization-associated anionic peroxidase 1-like	00940///Phenylpropanoid biosynthesis
101251197	‘LOC101251197’	0.3	2.1	2.7	0.04	4-Coumarate--CoA ligase	00130///Ubiquinone and other terpenoid-quinone biosynthesis+++00940///Phenylpropanoid biosynthesis
112940333	‘LOC112940333’	19.3	96.0	2.4	0.02	Lignin-forming anionic peroxidase-like	00940///Phenylpropanoid biosynthesis
101266208	‘LOC101266208’	15.4	49.6	1.8	0.01	4-Coumarate--CoA ligase-like 9	00130///Ubiquinone and other terpenoid-quinone biosynthesis+++00940///Phenylpropanoid biosynthesis
101262919	‘LOC101262919’	32.1	104.0	1.8	0.03	Cytochrome P450 CYP73A100	00130///Ubiquinone and other terpenoid-quinone biosynthesis+++00940///Phenylpropanoid biosynthesis+++00941///Flavonoid biosynthesis+++00945///Stilbenoid, diarylheptanoid and gingerol biosynthesis
101244162	‘LOC101244162’	32.0	101.4	1.7	0.01	Peroxidase 51	00940///Phenylpropanoid biosynthesis
544293	‘cevi16’	362.1	1086.4	1.7	0.03	Peroxidase	00940///Phenylpropanoid biosynthesis
101253556	‘LOC101253556’	8.5	25.6	1.7	0.00	Agmatine hydroxycinnamoyltransferase 1-like	00940///Phenylpropanoid biosynthesis+++00941///Flavonoid biosynthesis+++00945///Stilbenoid, diarylheptanoid and gingerol biosynthesis
101257228	‘LOC101257228’	332.1	754.7	1.3	0.00	Peroxidase 51	00940///Phenylpropanoid biosynthesis
101256743	‘LOC101256743’	10.8	23.0	1.2	0.00	Caffeoylshikimate esterase	00940///Phenylpropanoid biosynthesis
544249	‘hqt’	44.3	91.0	1.1	0.03	Hydroxycinnamoyl CoA quinate transferase	00940///Phenylpropanoid biosynthesis+++00941///Flavonoid biosynthesis+++00945///Stilbenoid, diarylheptanoid and gingerol biosynthesis
101265187	‘LOC101265187’	218.6	414.7	1.0	0.04	Caffeoyl-CoA O-methyltransferase-like	00940///Phenylpropanoid biosynthesis+++00941///Flavonoid biosynthesis+++00945///Stilbenoid, diarylheptanoid and gingerol biosynthesis
101267473	‘LOC101267473’	15.9	6.5	− 1.2	0.04	Peroxidase 64	00940///Phenylpropanoid biosynthesis
101252368	‘LOC101252368’	24.9	10.1	− 1.2	0.01	Peroxidase 5	00940///Phenylpropanoid biosynthesis
101263035	‘LOC101263035’	297.8	111.0	− 1.3	0.01	Peroxidase 47	00940///Phenylpropanoid biosynthesis
101055589	‘AT3'	5.5	1.5	− 1.8	0.00	AT3 protein	00940///Phenylpropanoid biosynthesis+++00941///Flavonoid biosynthesis+++00945///Stilbenoid, diarylheptanoid and gingerol biosynthesis

## Discussion

Numerous microorganisms inhabit the soil, including soil-borne pathogens, which can cause serious damage to crops. In the present study, we investigated the efficacy of FBP and its effects on plant immunity. FBP was effective in controlling bacterial wilt disease in 10 plant families, including Solanaceae plants, other than tomatoes, and had a concentration-dependent effect on bacterial canker in tomatoes. Furthermore, FBP treatment promoted the lignification of Solanaceae plants as a factor in the resistance mechanism.

Wilt symptoms in plants caused by *Cmm* differ from those in *Rps*. *Cmm* multiplies in the xylem and destroys cells using cell wall-degrading enzymes [18]. Conversely, accumulation of phenolic compounds, lignification of vessels, and callose deposition have been observed in *Cmm*-resistant tomato varieties [19]. This suggests that cell wall strengthening is a common form of resistance to these pathogens. Our results suggest that FBP induce plant immunity to control *Cmm* without affecting its toxicity or virulence, similar to its effect on *Rps*, and further suggest that lignin accumulation in the roots is one of its effects [8]. Controlling bacterial wilt disease and bacterial canker of tomato by enhancing plant resistance has been reported with acibenzorlar S-methyl (ASM) and β-aminobutyric acid (BABA) [20–22]. ASM- and BABA-treated plants upregulate phenylpropanoid pathway genes and increase the accumulation of compounds associated with cell wall strength, such as suberin and lignin [23, 24]. ASM, a precursor of salicylic acid, induces systemic acquired resistance by upregulating the expression of pathogenesis-related (*PR*) gene families in plants [25]. Although FBP does not contain the compounds mentioned above, the fact that the *SlPR1* gene was significantly upregulated by FBP treatment in tomato (data not shown) suggests that plant disease resistance is stimulated by a similar pathway. Plant elicitor peptide 1 (Pep1), a plant-derived damage-associated molecular pattern (DAMP), reportedly induces lignin in plants [26]. Although Pep is widely conserved in higher plants, low homology of peptide sequences among families limits the effects of Pep treatment on the family to which the species belongs [27]. Dozens of plant species are used as materials for FBP, and plant fragments produced during the fermentation process may act as elicitors. The results show that FBP treatment induces lignin in Solanaceae roots; however, as FBP do not contain Solanaceae plants, whether DAMP-induced signaling occurs remains to be investigated. Here, FBP was found to induce lignin formation, which acts as a physical barrier. Some species treated with 0.5% FBP appeared smaller than controls (Fig. S1). However, because we did not collect quantitative growth measurements (e.g. fresh/dry weight, height, leaf area) or assay TOR signalling, the present data cannot establish statistically significant growth inhibition. The FBP under investigation contains sugars, including glucose and fructose, as one of its major constituents [8]. Sugars modulate TOR signalling in a concentration‑dependent manner, and thus we hypothesize that FBP‑derived sugars at certain concentrations could affect growth via immune activation or stress responses [28, 29]. The experiment will entail the quantification of sugar content, in addition to the performance of concentration–response growth assays and assays of TOR activity. This is the first report of lignin-inducing fermentation products and fertilizers, and the first report of a substance that induces bacterial wilt disease control in a host other than Solanaceae plants. Further work will be conducted to investigate pathogen localization in the roots and the effects of FBP treatment on other stresses.

### Limitations

The relative importance of our findings in disease control needed to be determined. Moreover, caution should be exercised when drawing conclusions and applying them to other plant families. Further studies should investigate changes in lignin levels and biosynthesis genes at different times, changes in lignin levels with FBP treatment in plants other than Solanaceae, and pathogen localization in roots. As correlations among FBP, lignin, and control of bacterial wilt disease have not been demonstrated, future analysis using defective strains is required.

## Electronic Supplementary Material

Below is the link to the electronic supplementary material.

Supplementary Material 1

Supplementary Material 2

## Data Availability

Not applicable.

## Abbreviations

FBP: Fermented botanical product
Rps: Ralstonia pseudosolanacearum
KEGG: Kyoto Encyclopedia of Genes and Genomes
DEG: Differentially expressed genes
Cmm: *Clavibacter michiganensis* subsp. *michiganensis*
PAL: Phenylalanine ammonia lyase
PR: Pathogenesis-related
ASM: Acibenzorlar *S*-methyl (ASM)
BABA: β-Aminobutyric acid
Pep1: Plant elicitor peptide 1
DAMPs: Damage-associated molecular patterns
