# Supplementary material for: Fermented botanical product supports lignin development and suppresses soil-borne pathogens
Source: BMC Res Notes. 2026 Feb 17;19:129. doi: 10.1186/s13104-026-07716-7 (PMC13015045; doi:10.1186/s13104-026-07716-7)
Supplement: Supplementary file 1 — Supplementary Material 1 [file 13104_2026_7716_MOESM1_ESM.docx]

**RNA isolation and sequencing (Supplementary Methods — detailed)**

The leaves and roots of tomato plants treated with water or FBP for 2 days were collected and homogenized in liquid nitrogen. Total RNA was extracted using NucleoSpin® RNA (Macherey-Nagel, Germany) according to the manufacturer’s instructions. cDNA library construction and sequencing were performed by BGI Genomics Co., Ltd. Total RNA from each sample was used for polyA-based selection using oligo dT beads for mRNA enrichment. mRNA was fragmented, and single-stranded cDNA was generated using random N6-primed reverse transcription, followed by double-stranded cDNA synthesis with dUTP. Synthesized cDNA was subjected to end repair and then 3’ adenylated. Adaptors were ligated to the ends of the 3’-adenylated cDNA fragments. Prior to PCR amplification, the dUTP-labeled strand was selectively degraded by Uracil-DNA Glycosylase, and the remaining strand was amplified to generate a cDNA library suitable for sequencing. The PCR product was denatured by heating, and single-stranded DNA was cyclized using splint oligo-and DNA ligases. DNA nanoballs were synthesized using single-stranded DNA as the template, and sequenced using the DNBSEQ platform. Clean reads were obtained from the raw data by removing reads containing the adaptor, with N > 5%, and of low-quality. Filtered clean reads were aligned to the reference and genome sequences of *S. lycopersicum* (NCBI GenBank resource: GCF_000188115.4_SL3.0) using HISAT [**14**]. After alignment, gene quantification and other analyses were performed based on gene expression. Data were analyzed using the Dr. Tom network platform of the BGI (<http://report.bgi.com>). Kyoto Encyclopedia of Genes and Genomes (KEGG) pathway descriptions were searched for differentially expressed genes (DEG) belonging to the phenylpropanoid pathway; the corresponding genes were extracted with a threshold of absolute log2 ratio ≥ 1 and q value ≤ 0.05.
