# Supplementary material for: Fermented botanical product supports lignin development and suppresses soil-borne pathogens
Source: BMC Res Notes. 2026 Feb 17;19:129. doi: 10.1186/s13104-026-07716-7 (PMC13015045; doi:10.1186/s13104-026-07716-7)
Supplement: Supplementary file 2 — Supplementary Material 2 [file 13104_2026_7716_MOESM2_ESM.docx]

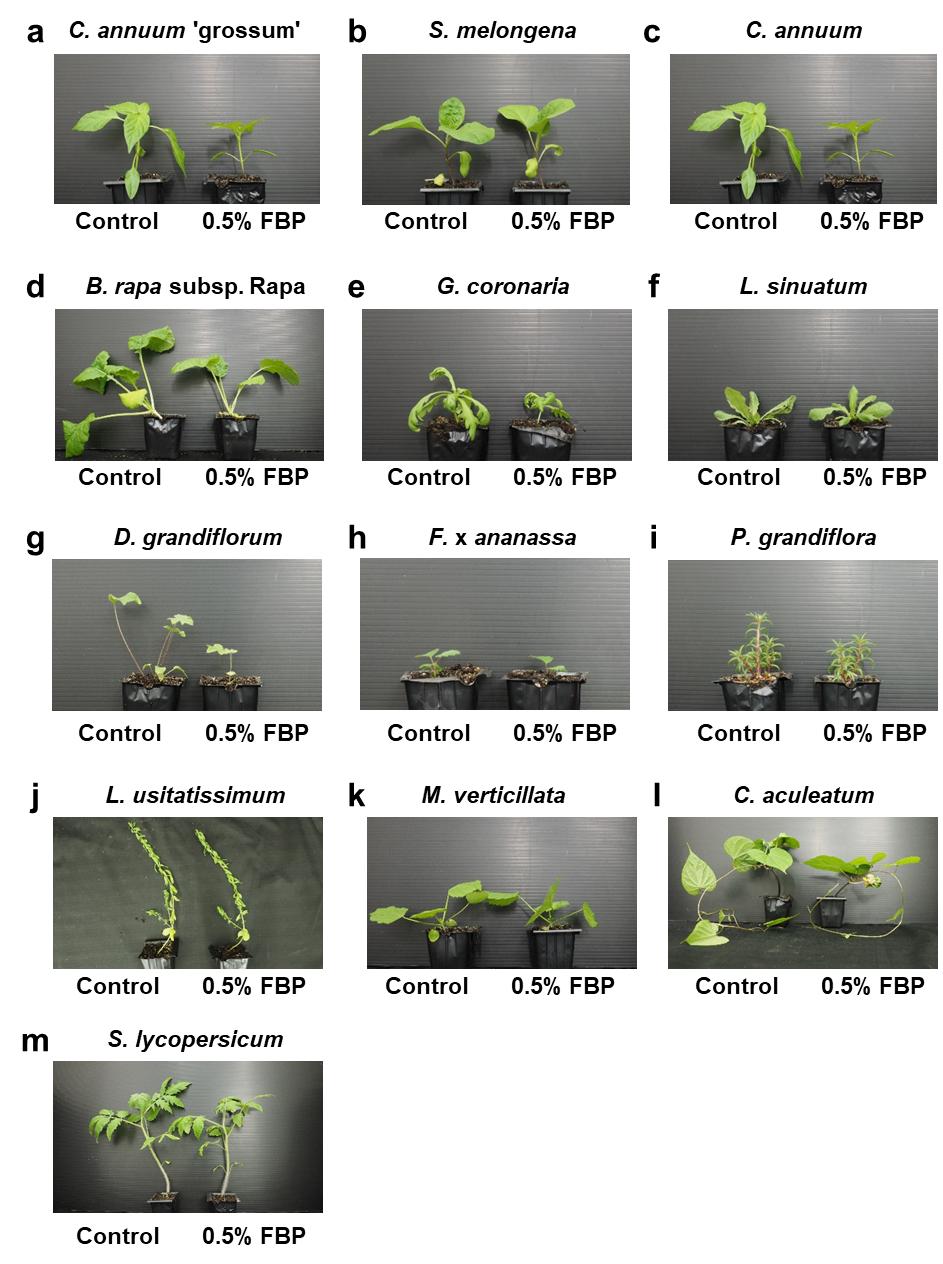


**Figure.S1** (a)-(m) Appearance of plants 14 or 21 days after water or FBP soaking treatment. Roots were cut and grown after the treatment.
